# Are Leukaemic Stem Cells Restricted to a Single Cell Lineage?

**DOI:** 10.3390/ijms21010045

**Published:** 2019-12-19

**Authors:** Geoffrey Brown, Lucía Sánchez, Isidro Sánchez-García

**Affiliations:** 1 School of Biomedical Sciences, Institute of Clinical Sciences, University of Birmingham, Birmingham B15 2TT, UK; 2 School of Law, University of Salamanca, 37007 Salamanca, Spain; id00698454@usal.es; 3 Experimental Therapeutics and Translational Oncology Program, Instituto de Biología Moleculary Celular del Cáncer, CSIC/Universidad de Salamanca, 37007 Salamanca, Spain; isg@usal.es; 4 Institute of Biomedical Research of Salamanca (IBSAL), 37007 Salamanca, Spain

**Keywords:** leukaemia, stem cells, haematopoiesis, lineage decision making, oncogenes, cancer

## Abstract

Cancer-stem-cell theory states that most, if not all, cancers arise from a stem/uncommitted cell. This theory revolutionised our view to reflect that cancer consists of a hierarchy of cells that mimic normal cell development. Elegant studies of twins who both developed acute lymphoblastic leukaemia in childhood revealed that at least two genomic insults are required for cancer to develop. These ‘hits’ do not appear to confer a growth advantage to cancer cells, nor do cancer cells appear to be better equipped to survive than normal cells. Cancer cells created by investigators by introducing specific genomic insults generally belong to one cell lineage. For example, transgenic mice in which the LIM-only 2 (*LMO2*, associated with human acute T-lymphoblastic leukaemia) and *BCR*-*ABL*^p210^ (associated with human chronic myeloid leukaemia) oncogenes were active solely within the haematopoietic stem-cell compartment developed T-lymphocyte and neutrophil lineage-restricted leukaemia, respectively. This recapitulated the human form of these diseases. This ‘hardwiring’ of lineage affiliation, either throughout leukaemic stem cell development or at a particular stage, is different to the behaviour of normal haematopoietic stem cells. While normal cells directly commit to a developmental pathway, they also remain versatile and can develop into a terminally differentiated cell that is not part of the initial lineage. Many cancer stem cells do not have this versatility, and this is an essential difference between normal and cancer stem cells. In this report, we review findings that support this notion.

## 1. Introduction

The precise cellular origin of a cancer, and the ability of the underlying cell to differentiate dictate the course of overt disease. This, in turn, influences how well patients respond to their treatment. The specific type of cell in which a cancer originates, the cancer-initiating cell (CIC), has been of longstanding interest to scientists and clinicians. Even now, the identity of this cell is unclear in some cancers. In the late 1990s, John Dick and his colleagues proposed cancer-stem-cell theory on the basis of evidence from studies of acute myeloid leukaemia (AML). They stated that most, if not all, cancers arise from genetic lesions involving a tissue-specific stem cell [1]. In the case of AML, the leukaemia-initiating cell (LIC) was a pluripotent haematopoietic stem cell (HSC). John Dick’s proposal was keeping with the view that HSCs and their progeny (haematopoietic progenitor cells, HPCs) were prime targets for malignant transformation due to their inherent properties of self-renewal, long life, and proliferative potential. To this day, investigators also view mature B and T lymphocytes as targets for malignancy by virtue of their proliferative potential [2].

There are two key cells with regard to the development of cancer, CICs and cancer stem cells (CSCs). These cells are often confused. Some 100 years ago, Boveri observed chromosomal abnormalities in cancer cells [3]. Investigators identified further lesions in the 1950s and 1960s, leading to the view that the underlying driver of the development of cancer was an insult to the genome. Around this time, cancer modelling also began to include the concept of cancer initiation (preneoplasia) in addition to overt disease (malignancy). Subsequently, elegant studies of identical twins who had both developed acute lymphoblastic leukaemia (ALL) in childhood revealed that at least two insults to the genome were required for the development of leukaemia [4]. This was in part due to the intraplacental sharing of a preleukaemic clone containing an identical chromosomal translocation. However, disease occurrence is asynchronous, and therefore requires one prenatal and one (or more) postnatal genomic ‘hits’. The first ‘hit’ converts the LIC, thought to be a B-lymphocyte-committed progenitor in the case of ALL (see below), into a dormant preleukaemic cell. A further ‘hit’ then converts this cell into a leukaemic stem cell (LSC) that gives rise to and sustains the disease. 

## 2. Nature of Cancer Stems Cells and Their Progeny

A clear picture of the behaviour of LICs/CICs and LSCs/CSCs is important to finding better treatments for malignancies. This is because LSCs are mostly responsible for untreatable disease relapses in AML, and CSCs underlie carcinoma relapse and metastasis. The elimination of CSCs might provide a means to find a cure for carcinomas that are difficult to treat. However, we do not currently have a clear picture of how two or more ‘hits’ transform a normal stem cell into a cancer cell. For example, we do not know if there is a difference between LIC and normal HSC behaviour in childhood ALL. Analysis of leukaemic cells at the time of patient presentation does not provide direct insight into the nature of LICs because the disease process begins many years prior, and the mutational burden is then too high.

CSCs constitute a small fraction of a patient’s cancer cells. The percentage ranges from very small to up to 25% for different types of cancers [5,6,7], meaning that more mature progeny form the bulk of a patient’s cancer cells. Investigations of HSCs and haematopoiesis have provided the current paradigm of normal stem-cell development and the nature of their progeny. Many longstanding and classical models of haematopoiesis depict a binary cell-fate tree in which the progeny of HSCs and HPCs progressively restrict their developmental options to give rise to end-cell types (see below). Investigators also use this type of model to describe the development of other tissue-specific stem cells. Human AML is composed of a hierarchy of cells that originate from a transformed HSC [8] that has differentiated to variable degrees to make up the bulk of a patient’s leukaemic cells. 

The behaviour of the bulk of a patient’s cancer cells might elucidate the difference between normal stem cells and CSCs. An early view on the nature of cancer cells arose from the first descriptions of patients with leukaemia by Bennet and Virkow. They reported a high density of white cells in blood and concluded that leukaemic cells divide extensively and/or quickly, and that the abundance of cells might be a response to infection. Rapid cell division at diagnosis is rare because many cancers are indolent, meaning they take time to become evident. The combined ‘hits’, therefore, do not necessarily confer a growth advantage to CICs/CSCs. Though cancers originate from a single transformed cell, the progeny are dynamic and undergo clonal evolution that has major implications for curing advanced cancers. Despite their diversity, cancer cells do not appear to be better equipped to survive. This was shown in a study where cancer subclones seldom demonstrated survival advantages over one another at the time of patient diagnosis [8]. The differentiation of cancer cells is often perturbed, which has led to the view that there is a skewing of cellular controls to favour proliferation over maturation. However, given that there are numerous factors controlling proliferation and maturation within cells [9,10], a proliferation increase at the expense of maturation is perhaps not a hallmark feature of cancer. Strikingly, leukaemic and carcinoma cells, including those arising from a stem cell, generally belong to one cell lineage. The following review examines whether this is a cardinal feature of cancer.

## 3. Leukaemic Cells Belong to One Lineage

The nature of a patient’s cells in chronic myeloid leukaemia (CML) clearly illustrates that an LSC can ‘dump’ cells down one lineage pathway. CML was shown to be a disease of HSCs by Fialkow, who used cells from female patients and X-linked glucose-6-phosphate to reveal that a wide spectrum of blood cell types belong to a leukaemic clone [11]. Despite this finding, patients’ cells were substantially restricted to the neutrophil pathway. Similarly, acute erythroid leukaemia is an HSC disease, and ≥80% of bone marrow cells are erythroid precursors [12]. 

Cell surface markers, particularly those identified by monoclonal antibodies, have provided a vital tool to determine the precise nature of leukaemic cells. Owing to these markers, an understanding of cell-lineage affiliation has advanced far more in leukaemia than in some other cancers. However, for some human leukaemia types, the common belief about a cell of origin is at odds with experiment evidence that supports earlier HSC origin. As mentioned above, ALL arises from a B-lymphocyte-committed progenitor [13]. In contrast, HSC-like cells that express marker CD34^+^ and lack B-lineage markers recreate the disease in mice [14]. We view acute promyelocytic leukaemia (APL) as a myeloid progenitor malignancy [15], with its hallmark feature being a t(15;17) chromosomal translocation giving rise to the PML-RARα oncoprotein. The translocation and oncoprotein are both present in CD34^+^CD38^−^ HSC-like cells in patients with APL [16]. It has been a longstanding belief that chronic lymphocytic leukaemia (CLL) is a malignancy of small B-lymphocytes. However, abnormal expression of lymphoid genes in patients’ HSCs, and the fact that HSCs purified from patients with CLL produce a high number of polyclonal B-cell progenitors, indicate an HSC origin [17].

Coupled to the assertion that leukaemic cells belong to one cell lineage, as defined by histological features and immunological markers, is that unique chromosomal abnormalities are also present. As stated above, the bulk of CML patients’ cells are relatively mature neutrophils. Hungerford and Novell described a specific chromosomal abnormality that is associated with CML—a small version of chromosome 21, termed the Philadelphia chromosome [18]. Since this discovery, the development of a high-resolution technique for chromosomal analysis and fluorescence in situ hybridisation (FISH) has led to the discovery of many other chromosomal abnormalities that result in hybrid genes consisting of portions of two different genes. In some cases, multiple different types of cancer express the same hybrid gene, indicating that these genes can play a role in the cancers of different tissue types. Like CML, some chromosomal abnormalities are specifically associated with the histological subtype of a malignancy, such as those seen in non-Hodgkin’s lymphoma. Specifically, a translocation between chromosomes 18 and 14 is seen in patients with follicular lymphoma, and a translocation between chromosomes 8 and 14 is seen in patients with small-noncleaved-cell and large immunoblastic lymphoma [19]. Chromosomal translocations between chromosomes 11 and 14, 11 and 13, and 7 and 11 activate the *LMO2* gene, the product of which is a specific marker of T-cell ALL (T-ALL) [20]. A translocation between chromosomes 12 and 21, creating the *ETV6*-*RUNX1* fusion gene, is associated with B-cell ALL (B-ALL) [21].

**Figure 1:**
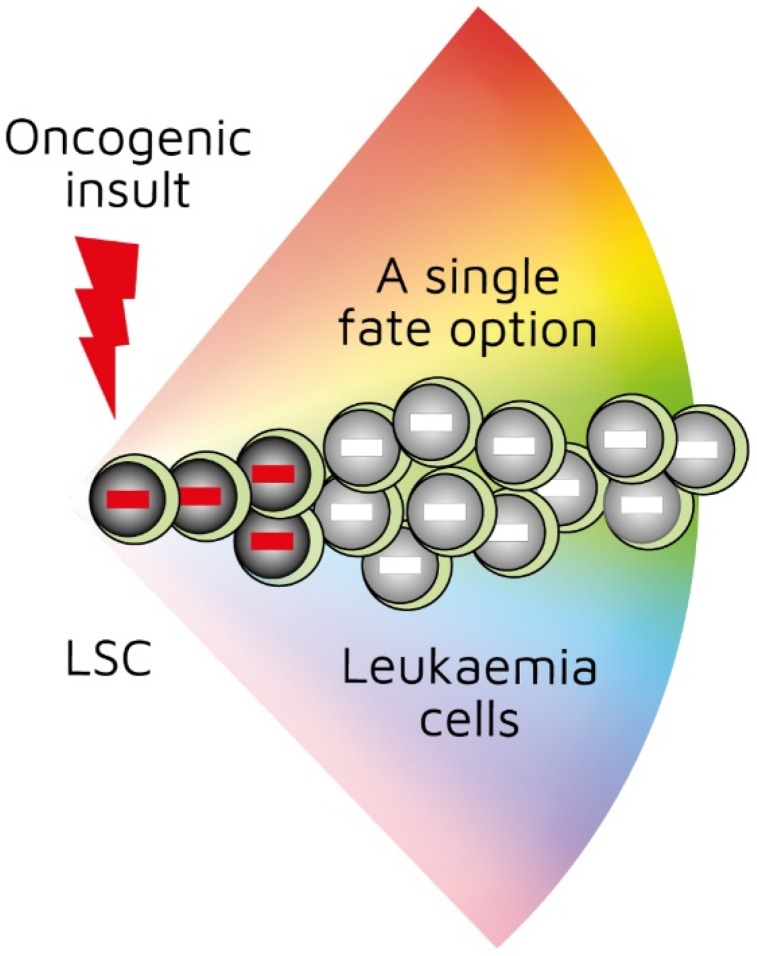
First oncogenic insult restricts leukaemic stem cells to a single differentiation pathway. *BCR*-*ABL*^p190^, *LMO2*, and *BCR*-*ABL*^p210^ oncogenes play a role in human B-lymphocyte, acute T-lymphoblastic, and chronic myeloid leukaemia, respectively. Studies of transgenic mice in which investigators used *Sca*-*1* promotor to restrict oncogene expression to haematopoietic stem cells showed that oncogenes initiated leukaemia development and recapitulated lineage-restricted human disease.

Investigators have been able to reproduce in mice the genotype–phenotype associations found in some human leukaemias by restricting the expression of a specific oncogene to the stem-/progenitor-cell compartment. Though leukaemia arises in a primitive cell, leukaemic cells belong to a particular cell lineage. Examples of this association in haematopoietic malignancies include *BCR*-*ABL*^p210^ in CML [22], *MAFB* in multiple myeloma [23], and *BCL6* in B-cell neoplasia [24]. Examples of this association in solid tumours include *EWS*-*FL1*-*1* in Ewing sarcoma [25] and *SYT*-*SSX2* in synovial sarcoma [26]. In these instances, a specific genetic insult to a stem/progenitor cell is associated with a particular cancer. The *BCR*-*ABL*^p190^, *LMO2*, and *BCR*-*ABL*^p210^ oncogenes play a role in human B-lymphocyte, acute T-lymphoblastic, and chronic myeloid leukaemia, respectively. In transgenic mice in which the expression of *BCR*-*ABL*^p190^, *LMO2*, and *BCR*-*ABL*^p210^ was restricted to HSCs via the *Sca-1* promotor, carcinogenesis was initiated by the oncogenes, and the resultant cancer recapitulated lineage-restricted human disease [27,28,29]. In transgenic mice, the oncogene is solely active within LICs/LSCs and is therefore not essential for the survival and/or proliferation of more mature lineage-affiliated leukaemic cells. An interpretation of these findings is that the oncogene ‘hardwires’ lineage affiliation either throughout or at a particular stage of LSC development, thus restricting the leukaemic cells to that pathway (Figure 1) [30]. This may occur via the oncogene-mediated priming of the epigenome in cells to adopt a single cell lineage [29,30].

While we argue that specific oncogenes/genomic insults to HSCs give rise to a particular lineage-restricted type of leukaemia, there are some caveats to extending this assertion to other types of cancer. Leukaemias could be unique in their specific genomic/epigenetic insults that serve to drive the transformed HSC along a particular developmental pathway. In addition, a specific insult/chromosomal abnormality is not seen in all cancers. This is especially true of solid tumours. We argue that some leukaemia types have an earlier HSC origin than traditionally thought, but that a lineage-committed progenitor cell may be the origin of some solid tumours. In this case, lineage affiliation is equated to the cell of origin, whereby the cell of origin dedifferentiates to regain stemness while retaining a close lineage affiliation. An argument against this view is that committed epithelial cells can give rise to malignant squamous cell carcinomas despite the absence of an oncogene to revert these cells to a stem-cell-like state [31]. However, it does seem that stem cells are usually the origin of ‘successful’ squamous malignancies.

There are malignancies in which the simultaneous expression of cell surface markers of different cell types confers a mixed lineage status. Coexpression of markers belonging to at least two lineages is seen in mixed-phenotype acute leukaemia (MPAL). This is a rare subgroup of acute leukaemia (2%–5%) in which cells express myeloid and B- or T-lymphoid markers, or myeloid, B-, and T-lymphoid markers together. MPAL might seem to contradict the oncogene-driven ‘hardwiring’ of HSCs to a cell lineage. However, our understanding of MPAL is still very limited because the causative cells are of ambiguous lineage and origin. It is not known whether it is more effective to treat MPAL patients with acute myeloid or acute lymphoid regimens. The surface expression of lineage markers might not reliably define the predominant cell type in MPAL. Indeed, clinicians consider some cases of MPAL to be acute myeloid leukaemia at diagnosis, with the expression of lymphoid markers being due to inappropriate gene expression [32]. As mentioned above, *ETV6*-*RUNX1* is associated with B-ALL despite blast cells expressing myeloid markers [33,34]. The same applies to *BCR*-*ABL*^190^ in B-ALL [35]. We view both of these leukaemias as being primarily of a B-lineage restricted cell with aberrant gene expression. Other hybrid states include the epithelial–mesenchymal transition, whereby a polarised epithelial cell undergoes changes to assume a mesenchymal-cell phenotype. This is seen in cancer cells at the invasive front of a tumour, where they convert to a mesenchymal phenotype to spread to other organs. We consider this transition somewhat different to the malignant transformation of HSCs because the epithelial–mesenchymal transition occurs during development and adulthood as a means of generating more cells [36].

There are leukaemias and solid cancers in which malignant cells are clearly a mixture of more than one cell type. A striking example is the transformation of embryonic stem cells to give rise to teratomas consisting of multiple cell types. This is also the case for the transformation of induced pluripotent cells (iPSCs). iPSC-derived teratomas resemble benign teratomas that occur in mice and humans during early life [37]. We consider them ‘unsuccessful’ malignancies and excluded them from consideration with regard to lineage fixation. The classification and origin of spontaneous teratomas in humans is complex and unclear. Some types seem to arise from multiple defects during germline development [37]. As such, their development does not seem to be initiated by an oncogene-driven event. 

## 4. Do Normal Stem Cells Behave Differently?

**Figure 2:**
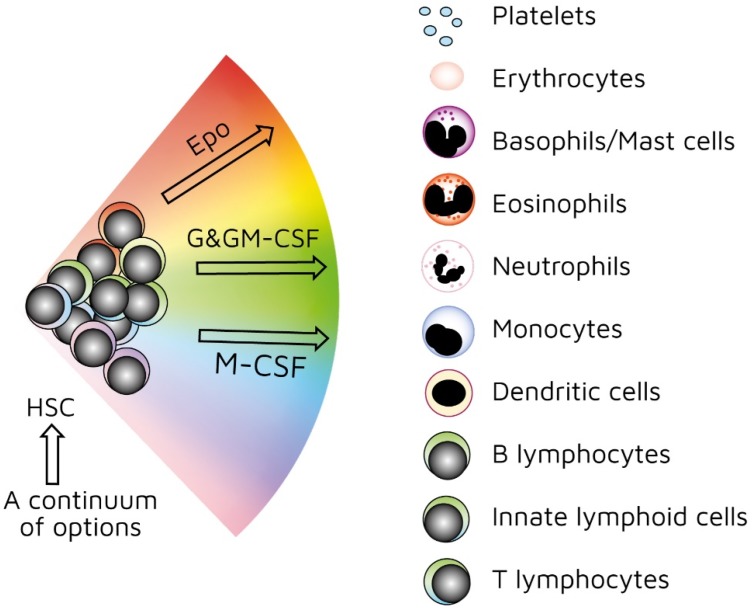
Continuum haematopoiesis model. In our model, haemopoietic stem cells (HSCs) are a mixture of cells with different lineage signatures (shown by different colours) that ‘choose’ a lineage from all options. HSCs and haematopoietic progenitor cells (HPCs) retain enough versatility to ‘step sideways’ into a different pathway. Erythropoietin (Epo), granulocyte colony-stimulating factor (G-CSF) and granulocyte/macrophage colony-stimulating factor (GM-CSF), and macrophage colony stimulating factor (M-CSF) can induce erythroid, neutrophil, and monocyte fates, respectively, within HSCs/HPCs.

As mentioned above, investigators have used treelike models for many years to describe the development of HSCs. In recent years, our view has radically changed. We previously viewed HSCs as a pluripotent population of cells. Branch points in treelike models of haematopoiesis dictate routes that individual HSCs follow towards each end-cell fate via a series of intermediate HPCs. The final step involves commitment to a single lineage. However, HSCs selectively express lineage-associated receptors on their surface: for erythropoietin (Epo) [38], macrophage colony-stimulating factor (M-CSF) [39,40,41], granulocyte colony-stimulating factor (G-CSF) [42], granulocyte/macrophage colony-stimulating factor (GM-CSF) [39], and FMS-like tyrosine kinase 3 ligand (Flt3L) [41]. In keeping with findings that support the initiation of lineage affiliation earlier than previously thought (Figure 2), recent studies have shown that, within HSCS, human adult bone-marrow CD34^+^ cells include pluripotent cells and cells with unipotent myeloid or erythroid potential. In this case, the downstream progeny of HSCs that we have routinely considered to be a single population of cells on the basis of cell surface markers could also be a mixture of cells with lineage signatures. This is true for the population of cells first described as early progenitors with lymphoid and myeloid potential (EPLM) [43]. RNA sequencing of single cells in a ‘primitive’ subpopulation of EPLM that lacked expression of markers Ly6D, SiglecH, and CD11c revealed that the subpopulation was composed of cells with a myeloid, dendritic cell, or lymphoid signature. Few cells had lymphoid and myeloid potential [44].

Our ‘developmental’ view of haematopoiesis is that an HSC selects one lineage from a continuum of all end-cell options, and then differentiates to that cell type (Figure 2). We provided evidence to support our viewpoint elsewhere [45,46,47]. Though the model shows that affiliation/commitment to a cell lineage can occur as early as during the HSC stage, haematopoiesis still remains dynamic. HSCs and HPCs can still ‘change their mind’ and adopt an alternative fate. Megakaryocyte-primed HSCs can ‘step sideways’ to erythropoiesis, as there is a ‘shared’ trajectory in these pathways due to a common dependence on transcription factor GATA-1 [48]. Investigators found that cells are able to move to the left or right of a chosen trajectory in the erythroid, granulocytic/macrophage, and lymphoid pathways. They established this by using the RNA sequencing of more than 1600 single HSCs and HPCs to construct expression maps [49].

With regard to HPCs, it was shown that mouse progenitor thymocytes that are well on their way to becoming T cells are not firmly restricted to this option—they can still give rise to macrophages, natural killer cells, and B-lymphocytes. The presence of an appropriate cytokine (e.g., M-CSF for macrophages) is essential to divert their fate in vitro (see below) [50]. Dispersal of HPCs from the bone marrow in a semisolid medium with the addition of particular growth factors (colony-stimulating factors) leads to the production of colonies containing various cell types depending on the added growth factor(s). We argued that the single cell giving rise to the colony ‘shuffles sideways’ to adopt various pathways because it is out of its normal environment with regard to the control of cell fate [46]. Single-cell analysis of mixed-lineage states has led to the proposal that bursts of alternative gene expression are an important feature of cell decision making, including the existence of bilineage states [51].

As mentioned above, the presence of M-CSF is required to derive macrophages from thymocyte progenitors. The selective expression of cytokine receptors by HSCs is relevant to how they ‘choose’ to follow a pathway of development. We formerly viewed all the above cytokines as survival and growth factors for cells that are committed to a certain cell lineage(s). We now know that they also instruct lineage choice. Epo commits multipotent HPCs to erythropoiesis [52]. M-CSF induces a myeloid lineage fate in HSCs [40], and a macrophage fate in granulocyte/macrophage progenitors [53]. G-CSF and GM-CSF induce the generation of neutrophils from granulocyte/macrophage progenitors (Figure 2) [53,54]. Flt3L drives the myeloid–lymphoid development of primitive mouse bone-marrow cells, suppressing the generation of megakaryocyte and erythroid progenitors [55]. Like normal haematopoietic cells, leukaemic cells (e.g., AML cells) depend on the presence of growth factors and an appropriate environment for their survival [56]. Therefore, we must assume growth factors are readily available. A supply of haematopoietic cytokines may continuously ‘shape’ the production of various types of blood cells to meet the overall demand of an organism. This may include diverting HSCs/HPCs towards a certain cell type to deal with particular circumstances or infectious agents [46]. For example, HSCs, rather than multipotent HPCs, drive erythropoiesis during chronic erythroid stress in Epo-transgenic mice. These mice demonstrate enhanced cell division, with HSCs exhibiting a committed erythroid progenitor profile [57]. 

## 5. Concluding Remarks

Given the above evidence, the first oncogenic insult to a tissue-specific stem cell initiates carcinogenesis by restricting lineage choices of LSCs and other CSCs, and directing their progeny into a single developmental pathway [30,58]. In this scenario, LSCs/CSCs and their progeny lack the versatility of HSCs/HPCs, which is an essential difference between normal and cancer stem cells. However, several components of this phenomenon are poorly understood. Specifically, the degree to which LSCs/CSCs are restricted is unknown, and, in other words, whether they are restricted from all other fate options. The ability to ‘unlock’ these cells from their fates should also be investigated. As stated above, we now know that Epo, M-CSF, G-CSF, GM-CSF, and Flt3L induce a specific cell lineage. We can use these cytokines to test whether they are able to redirect the differentiation of mouse LSCs in B-lymphocyte and T-lymphocyte leukaemias, and whether Epo can divert LSCs in myeloid leukaemia towards erythropoiesis. In addition, by dissecting how epigenetic priming plays a role in the risk and clinical course of leukaemia, we may be able to intervene by developing epigenetic modifiers. Unlike genetic changes, epigenetic changes can be manipulated in order to treat patients and even before a preleukaemic cell has evolved into leukaemia.

## References

[1] Dick J.E. (2008). Stem cell concepts renew cancer research. Blood.

[2] Salmon S., Seligman M. (1974). B cell neoplasia in man. Lancet.

[3] Wright N.A. (2014). Boveri at 100: Cancer evolution, from preneoplasia to malignancy. J. Pathol..

[4] Greaves M.F., Wiemels J., Ford A.M. (2003). Leukaemia in twins: Lessons in natural history. Blood.

[5] Bonnet D., Dick J.E. (1997). Human acute myeloid leukaemia is organised as a hierarchy that originates from a primitive hematopoietic cell. Nat. Med..

[6] Quintiana E., Shakleton M., Sabel M.S., Fullen D.R., Johnson T.M., Morrison S.J. (2008). Efficient tumour formation by single human carcinoma cells. Nature.

[7] Vicente-Duenas C., Perez-Caro M., Abullo-Jimenez F., Cobaleda C., Sanchez-Garcia I. (2009). Stem cell driven cancer: “hands-off” regulation of cancer development. Cell Cycle.

[8] Yates L.R., Campbell P.J. (2012). Evolution of the cancer genome. Nat. Rev. Genet..

[9] Drayson M.T., Michell R.H., Durham J., Brown G. (2001). Cell proliferation and CD11b expression are controlled independently during HL60 differentiation initiated by 1, 25α-dihydroxyvitamin D_3_ or all-*trans*-retinoic acid. Exp. Cell Res..

[10] Brown G., Hughes P.J., Michell R.H. (2003). Cell differentiation and proliferation-simultaneous but independent?. Exp. Cell Res..

[11] Fialkow P.J., Denman A.M., Jacobson R.J., Lowenthal M.N. (1978). Chronic myelocytic leukaemia: Origin of some lymphocytes from leukaemic stem cells. J. Clin. Investig..

[12] Kowal-Vern A., Mazzella F.M., Cotelingham J.D., Shrit M.A., Rector J.T., Schumacher H.R. (2000). Diagnosis andcharacterisation of acute erythroleukaemia subsets by determining the percentages of myeloblasts and proerythroblasts in 69 cases. Am. J. Hematol..

[13] Greaves M.F. (1999). Molecular genetics, natural history and the demise of childhood leukaemia. Eur. J. Cancer.

[14] Cox C.V., Blair A. (2005). A primitive cell origin for B-cell precursor ALL?. Stem Cell Rev..

[15] Grimwade D., Enver T. (2004). Acute promyelocytic leukaemia: Where does it stem from?. Leukemia.

[16] Edwards R.H., Wasik M.A., Finan J., Rodriguez R., Moore J., Kamoun M., Rennert H., Bird J., Nowell P.C., Salhany K.E. (1999). Evidence for early hematopoietic progenitor cell involvement in acute promyelocytic leukemia. Am. J. Clin. Pathol..

[17] Kikushige Y., Ishikawa F., Miyamoto T., Shima T., Urata S., Yoshimoto G., Mori Y., Iino T., Yamauchi T., Eto T. (2011). Self-renewing hematopoietic stem cell is the primary target in pathogenesis of human chronic lymphocytic leukemia. Cancer Cell.

[18] Nowell P., Hungerford D. (1960). A minute chromosome in human granulocytic leukaemia. Science.

[19] Yunis J.J., Oken M.M., Kaplan M.E., Ensrud K.M., Howe R.R., Theologides A. (1982). Distinctive chromosomal abnormalities in histologic subtypes of non-Hodgkin’s lymphoma. N. Eng. J. Med..

[20] Chang-Hoon N., Rabbits T.H. (2006). The role of LMO2 in development and in T cell leukaemia after chromosomal translocation or retroviral insertion. Mol. Ther..

[21] Shurtleff S.A., Buijs A., Behm F.G., Rubnitz J.E., Raimondi S.C., Hancock M.L., Chan G.C., Pui C.H., Grosveld G., Downing J.R. (1995). TEL/AML1 fusion resulting from a cryptic t(12;21) is the most common genetic lesion in pediatric ALL and defines a subgroup of patients with an excellent prognosis. Leukemia.

[22] Pérez-Caro M., Cobaleda C., González-Herrero I., Vicente-Dueñas C., Bermejo-Rodríguez C., Sánchez-Beato M., Orfao A., Pintado B., Flores T., Sánchez-Martín M. (2009). Cancer induction by restriction of oncogene expression to the stem cell compartment. EMBO J..

[23] Vicente-Dueñas C., Romero-Camarero I., González-Herrero I., Alonso-Escudero E., Abollo-Jiménez F., Jiang X., Gutierrez N.C., Orfao A., Marín N., Villar L.M. (2012). A novel molecular mechanism involved in multiple myeloma development revealed by targeting MafB to haematopoietic progenitors. EMBO J..

[24] Green M.R., Vicente-Dueñas C., Romero-Camarero I., Liu C.L., Dai B., González-Herrero I., García-Ramírez I., Alonso-Escudero E., Iqbal J., Chan W.C. (2014). Transient expression of Bcl6 is sufficient for oncogenic function and induction of mature B-cell lymphoma. Nat. Commun..

[25] Riggi N., Suvà M.L., De Vito C., Provero P., Stehle J.C., Baumer K., Cironi L., Janiszewska M., Petricevic T., Suvà D. (2010). EWS-FLI-1 modulates miRNA145 and SOX2 expression to initiate mesenchymal stem cell reprogramming toward Ewing sarcoma cancer stem cells. Genes Dev..

[26] Garcia C.B., Shaffer C.M., Alfaro M.P., Smith A.L., Sun J., Zhao Z., Young P.P., VanSaun M.N., Eid J.E. (2012). Reprogramming of mesenchymal stem cells by the synovial sarcoma-associated oncogene SYT-SSX2. Oncogene.

[27] Martín-Lorenzo A., Auer F., Chan L.N., García-Ramírez I., González-Herrero I., Rodríguez-Hernández G., Bartenhagen C., Dugas M., Gombert M., Ginzel S. (2018). Loss of Pax5 exploits Sca1-*BCR*-*ABL*^p190^ susceptibility to confer the metabolic shift essential for pB-ALL. Cancer Res..

[28] García-Ramírez I., Bhatia S., Rodríguez-Hernández G., González-Herrero I., Walter C., De Tena-Dávila S.G., Parvin S., Haas O., Woessmann W., Stanulla M. (2018). Lmo2 expression defines tumor cell identity during T-cell leukemogenesis. EMBO J..

[29] Vicente-Dueñas C., González-Herrero I., Sehgal L., García-Ramírez I., Rodríguez-Hernández G., Pintado B., Blanco O., Criado F.J.G., Cenador M.B.G., Green M.R. (2019). Dnm1 links *BCR*-*ABL*^p210^ to epigenetic tumour stem cell priming in myeloid leukaemia. Leukaemia.

[30] Vicente-Dueñas C., Hauer J., Cobaleda C., Borkhardt A., Sánchez-García I. (2018). Epigenetic priming in cancer initiation. Trends Cancer.

[31] Sánchez-Danés A., Blanpain C. (2018). Deciphering the cells of origin of squamous cell carcinomas. Nat. Rev. Cancer.

[32] Kim H.J. (2016). Mixed-phenotype acute leukemia and beyond. Blood Res..

[33] Abdelhaleem M. (2007). Frequent but nonrandom expression of myeloid markers on de novo childhood acute lymphoblastic leukemia. Exp. Mol. Pathol..

[34] Iijima K., Sugita K., Inukai T., Goi K., Tezuka T., Uno K., Sato H., Kagami K., Nakazawa S. (2000). Expression of thrombopoietin receptor and its functional role in human B-precursor leukemia cells with 11q23 translocation or Philadelphia chromosome. Leukemia.

[35] Pérez-Caro M., Gutierrez-Cianca N., González-Herrero I., López-Hernández I., Flores T., Orfao A., Sánchez-Martín M., Gutiérrez-Adán A., Pintado B., Sánchez-García I. (2007). Sustained leukaemic phenotype after inactivation of *BCR*-*ABL*^p190^ in mice. Oncogene.

[36] Kalluri R., Weinberg R.A. (2009). The basics of epithelial-mesenchymal transition. J. Clin. Investig..

[37] Cunningham J.J., Ulbright T.M., Pera M.F., Looijenga L.H. (2012). Lessons from human teratomas to guide development of safe stem cell therapies. Nat. Biotechnol..

[38] Shinjo K., Takeshita A., Higuchi M., Ohnishi K., Ohno R. (1997). Erythropoietin receptor expression on human bone marrow erythroid precursor cells by a newly-devised quantitative flow-cytometric assay. Br. J. Haematol..

[39] Kondo M., Scherer D.C., Miyamoto T., King A.G., Akashi K., Sugamura K., Weissman I.L. (2000). Cell-fate conversion of lymphoid-committed progenitors by instructive actions of cytokines. Nature.

[40] Mossadegh-Keller N., Sarrazin S., Kandalla P.K., Espinosa L., Stanley E.R., Nutt S.L., Moore J., Sieweke M.H. (2013). M-CSF instructs myeloid lineage fate in single haematopoietic stem cells. Nature.

[41] Mooney C.J., Cunningham A., Tsapogas P., Toellner K.M., Brown G. (2017). Selective expression of flt3 within the mouse hematopoietic stem cell compartment. Int. J. Mol. Sci..

[42] Liu F., Poursine-Laurent J., Link D.C. (2000). Expression of the G-CSF receptor on hematopoietic progenitor cells is not required for their mobilisation by G-CSF. Blood.

[43] Balciunaite G., Ceredig R., Massa S., Rolink A.G. (2005). A b220 + cd117 + cd19-hematopoietic progenitor with potent lymphoid and myeloid developmental potential. Eur. J. Immunol..

[44] Alberti-Servera L., Von Muenchow L., Tsapogas P., Capoferri G., Eschbach K., Beisel C., Ceredig R., Ivanek R., Rolink A. (2017). Single-cell RNA sequencing reveals developmental heterogeneity among early lymphoid progenitors. EMBO J..

[45] Ceredig R., Rolink A.G., Brown G. (2009). Models of hematopoiesis: Seeing the wood for the trees. Nat. Rev. Immunol..

[46] Brown G., Tsapogas P., Ceredig R. (2018). The changing face of hematopoiesis: A spectrum of options is available to stem cells. Immunol. Cell Biol..

[47] Brown G., Ceredig R. (2019). Modelling the hematopoietic landscape. Front. Cell Dev. Biol..

[48] Psaila B., Mead A.J. (2019). Single-cell approaches reveal novel cellular pathways for megakaryocyte and erythroid differentiation. Blood.

[49] Nestorowa S., Hamey F.K., Sala B.P., Diamanti E., Shepherd M., Laurenti E., Wilson N.K., Kent D.G., Gottens B. (2016). A single-cell resolution map of mouse hematopoietic stem and progenitor cell differentiation. Blood.

[50] Balciunaite G., Ceredig R., Rolink A.G. (2005). The earliest subpopulation of mouse thymocytes contains potent T, significant macrophage, and natural killer but no B-lymphocyte potential. Blood.

[51] Olsson A., Venkatasubramanian M., Chaudhri V.K., Aronow B.J., Salomonis N., Singh H., Grimes H.L. (2016). Single-cell analysis of mixed-lineage states leading to a binary cell fate choice. Nature.

[52] Grover A., Mancini E., Moore S., Mead A.J., Atkinson D., Rasmussen K.D., O’Carrol D., Jacobsen S.E., Nerlov C. (2014). Erythropoietin guides multipotent hematopoietic progenitor cells toward an erythroid fate. J. Exp. Med..

[53] Rieger M.A., Hoppe P.S., Smejkal B.M., Eitelhuber A.C., Schroeder T. (2009). Hematopoietic cytokines can instruct lineage choice. Science.

[54] Metcalf D., Burgess A.W. (1982). Clonal analysis of progenitor cell commitment of granulocyte or macrophage production. J. Cell Physiol..

[55] Tsapogas P., Swee L.K., Nusser A., Nuber N., Kreuzaler M., Capoferri G., Rolink H., Ceredig R., Rolink A. (2014). In vivo evidence for an instructive role of fms-like tyrosine kinase-3 (flt3) ligand in hematopoietic development. Haematologica.

[56] Griffin J.D., Lowenberg B. (1986). Clonogenic cells in acute myeloblastic leukaemia. Blood.

[57] Singh R.P., Grinenko T., Ramasz B., Franke K., Lesche M., Dahl A., Gassmann M., Chavakis T., Henry I., Wielockx B. (2018). Hematopoietic stem cells but not multipotent progenitors drive erythropoiesis during chromic erythroid stress in epo transgenic mice. Stem Cell Rep..

[58] Brown G., Sanchez-Garcia I. (2015). Is lineage decision-making restricted during tumoral reprograming of haematopoietic stem cells?. Oncotarget.

